# A Comprehensive Search of Non-Canonical Proteins in Non-Small Cell Lung Cancer and Their Impact on the Immune Response

**DOI:** 10.3390/ijms23168933

**Published:** 2022-08-11

**Authors:** Ehsan Irajizad, Johannes F. Fahrmann, James P. Long, Jody Vykoukal, Makoto Kobayashi, Michela Capello, Chuan-Yih Yu, Yining Cai, Fu Chung Hsiao, Nikul Patel, Soyoung Park, Qian Peng, Jennifer B. Dennison, Taketo Kato, Mei Chee Tai, Ayumu Taguchi, Humam Kadara, Ignacio I. Wistuba, Hiroyuki Katayama, Kim-Anh Do, Samir M. Hanash, Edwin J. Ostrin

**Affiliations:** 1 Department of Clinical Cancer Prevention, The University of Texas MD Anderson Cancer Center, 1515 Holcombe Blvd., Houston, TX 77030, USA; 2 Department of Biostatistics, The University of Texas MD Anderson Cancer Center, 1515 Holcombe Blvd., Houston, TX 77030, USA; 3 Department of Basic Pathology, School of Medicine, Fukushima Medical University, Hikarigaoka, Fukushima 960-1247, Japan; 4 Department of Thoracic Surgery, Nagoya University, Nagoya 464-8601, Japan; 5 Department of Translational Molecular Pathology, The University of Texas MD Anderson Cancer Center, 1515 Holcombe Blvd., Houston, TX 77030, USA; 6 Division of Molecular Diagnostics, Aichi Cancer Center, Nagoya 464-8601, Japan; 7 Division of Advanced Cancer Diagnostics, Nagoya University Graduate School of Medicine, Nagoya 464-8601, Japan; 8 Departments of General Internal Medicine, The University of Texas MD Anderson Cancer Center, 1515 Holcombe Blvd., Houston, TX 77030, USA

**Keywords:** noncanonical open reading frames, altORFs, neoantigens, autoantibodies, NSCLC

## Abstract

There is substantial interest in mining neoantigens for cancer applications. Non-canonical proteins resulting from frameshift mutations have been identified as neoantigens in cancer. We investigated the landscape of non-canonical proteins in non-small cell lung cancer (NSCLC) and their induced immune response in the form of autoantibodies. A database of cryptoproteins was computationally constructed and comprised all alternate open reading frames (altORFs) and ORFs identified in pseudogenes, noncoding RNAs, and untranslated regions of mRNAs that did not align with known canonical proteins. Proteomic profiles of seventeen lung adenocarcinoma (LUAD) cell lines were searched to evaluate the occurrence of cryptoproteins. To assess the immunogenicity, immunoglobulin (Ig)-bound cryptoproteins in plasmas were profiled by mass spectrometry. The specimen set consisted of plasmas from 30 newly diagnosed NSCLC cases, pre-diagnostic plasmas from 51 NSCLC cases, and 102 control plasmas. An analysis of LUAD cell lines identified 420 cryptoproteins. Plasma Ig-bound analyses revealed 90 cryptoproteins uniquely found in cases and 14 cryptoproteins that had a fold-change >2 compared to controls. In pre-diagnostic samples, 17 Ig-bound cryptoproteins yielded an odds ratio ≥2. Eight Ig-bound cryptoproteins were elevated in both pre-diagnostic and newly diagnosed cases compared to controls. Cryptoproteins represent a class of neoantigens that induce an autoantibody response in NSCLC.

## 1. Introduction

There is an increasing appreciation of the role of humoral immunity in immune surveillance, with findings of tumor infiltrating B lymphocytes being documented in numerous cancers [1,2]. The B-cell response occurs early during tumor development, resulting in the production of autoantibodies against tumor antigens [3,4,5]. Several strategies have been applied for the discovery of circulating autoantibodies in cancer, including the serological screening of cDNA expression libraries (SEREX) [6], recombinant arrays [7,8], and phage-display libraries [9]. Tumor cell lysate-derived protein arrays have been utilized to define autoantibody signatures [10,11]. A more global approach consists of utilizing mass spectrometry to identify circulating antigen–antibody complexes.

Recent studies have demonstrated that eukaryotic transcripts may encompass non-canonical alternate open reading frames (altORFs), resulting in proteins with altered subcellular localization signals or different biological activities [12]. Additionally, some transcripts contain short upstream open reading frames (uORFs) that have a well-described role in translational regulation [13]. A recent report indicated that 6.5% of MHC-bound peptides were derived from a non-canonical reading frame. These peptides originated from the frameshifted translation of protein-coding transcripts, and the resulting peptides were shown to be immunogenic to peripheral blood-derived mononuclear cells [14]. Another study incorporated transcriptomics, ribosomal profiling, and mass spectrometry to elucidate hundreds of shared and tumor-specific, non-canonical HLA-bound peptides [15]. A study of melanoma cell lines showed that the induction of IFNγ through depletion of tryptophan contributes to the immune recognition of melanoma cells through an aberrant peptidome [16]. However, to date, there have been no global searches for immunogenic non-canonical proteins in cancer.

In prior studies, we identified autoantibody signatures in lung cancer from samples collected at the time of diagnosis as well as samples collected one or more years preceding diagnosis [17,18,19]. These signatures consisted of proteins and peptides derived from the canonical ORFs. We hypothesized that, given widespread translational dysregulation in cancer [20,21], novel proteins derived from altORFs, pseudogenes, intronic regions, and other transcripts considered not to encode proteins represent a novel source of tumor antigens that can elicit an immune response resulting in autoantibodies. We designate these non-canonical proteins ‘cryptoproteins’. We first constructed a database of novel cryptoproteins with no homology to the canonical human peptidome that could be identified with a low false discovery rate using mass spectrometry-based proteomic analysis. We then applied this approach to demonstrate the occurrence of cryptoproteins in lung adenocarcinoma cell lines. Next, we identified circulating cryptoprotein–antibody complexes elevated in plasma samples from newly diagnosed non-small cell lung cancer (NSCLC) patients as well as plasma samples collected prior to diagnosis compared to controls.

## 2. Results

**The cryptoproteome of human lung adenocarcinoma cell lines**

**Figure 1:**
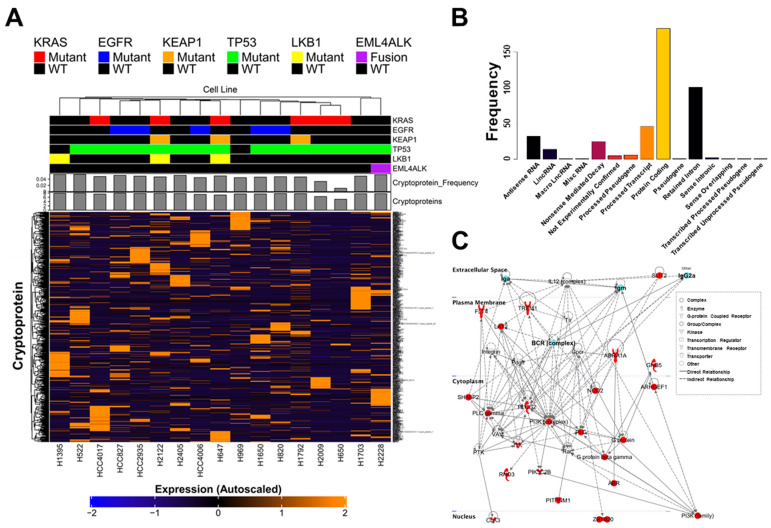
Cryptoproteins are widely detected in human lung adenocarcinoma cell lines: (**A**) A heatmap representation of cryptoprotein expression in lung adenocarcinoma cell lines with annotated driver mutation (KRAS, EGFR, KEAP1, TP53, LKB1, EML4/ALK) shows no association between mutational status and cryptoprotein production. (**B**) Cryptoproteins identified and quantified in lung adenocarcinoma cell lines. (**C**) Ingenuity pathway analyses of genes corresponding to the 420 cryptoproteins detected in lung adenocarcinoma cell lines revealed enrichment of inflammatory pathways with B-cell receptor and immunoglobulin families as central network nodes.

Using our customized pipeline and cryptoDB, we evaluated the cryptoproteome of 17 human lung adenocarcinoma cell lines previously profiled by mass spectrometry (Supplemental Table S1) [22,23]. A total of 420 cryptoproteins were identified with PSM ≥ 5 and that were quantifiable in two or more cell lines. The abundance of cryptoproteins was not associated with prevalent (*KRAS*, *EGFR*, *KEAP1*, *LKB1*, *TP53*) mutations commonly observed in lung adenocarcinomas (Figure 1A) [24]. Of the 420 quantified cryptoproteins, 183 (43.6%) were derived from protein-coding genes, 101 (24.0%) from retained introns, 46 (11.0%) from processed transcripts, 32 (7.6%) from antisense RNAs, 25 (6.0%) transcripts flagged as undergoing nonsense mediated decay, 14 (3.3%) from long intergenic noncoding RNA (lincRNA), 6 (1.4%) from processed pseudogenes, 5 (1.2%) from non-experimentally confirmed regions, two (<1%) from sense intronic, and one each (<1%) of macro-lincRNA, miscellaneous RNA, unspecified pseudogene, sense overlapping, transcribed processed pseudogene, and transcribed unprocessed pseudogene (Figure 1B).

Ingenuity pathway analysis of the 420 canonical gene names corresponding to the identified cryptoproteins revealed immune-centric networks, with the B-cell receptor and immunoglobulin (Ig) genes family being represented as central nodes (Figure 1C), consistent with previous findings [14,15,16].

**Circulating Ig-bound cryptoproteins in NSCLC**

**Figure 2:**
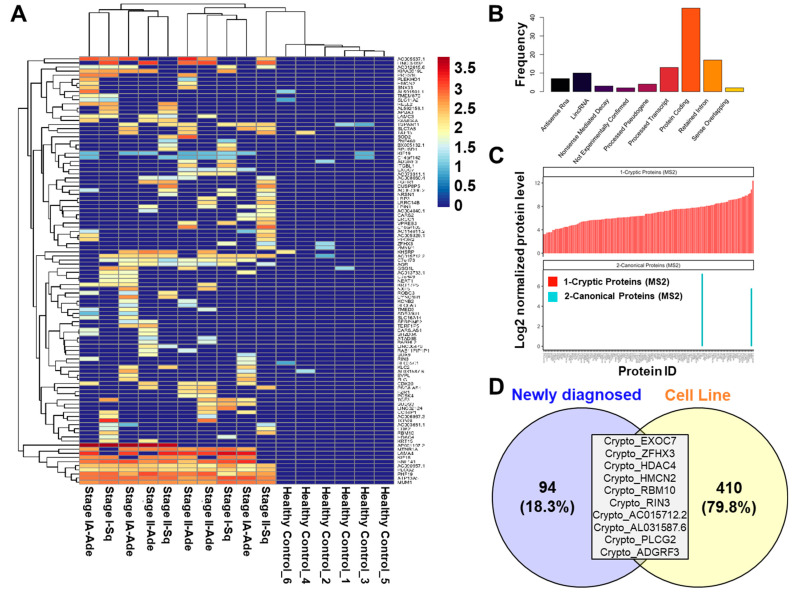
A unique Ig-bound cryptoprotein signature is detected in plasma from individuals newly diagnosed with NSCLC: (**A**) Heatmap depicting 104 Ig-bound cryptoproteins that are either uniquely identified or had a fold-change greater than 2 in cases compared to controls. Abbreviations: Sq, Squamous Cell Carcinoma; Adc, Adenocarcinoma. (**B**) Types of cryptoproteins that were elevated in the Ig-bound fraction of newly diagnosed lung cancer cases compared to controls. (**C**) Comparison of Ig-bound cryptoproteins and their canonical counterparts reveals near exclusivity of Ig reactivity against cryptoproteins. (**D**) Venn diagram illustrating overlap between cryptoproteins identified in lung adenocarcinoma cell lines with cryptoproteins that were elevated in the Ig-fraction of newly diagnosed lung cancer cases.

To expand upon our findings and determine the extent by which cryptoproteins were associated with a humoral response in the context of NSCLC, we performed the proteomic mass spectrometry-based analyses of Ig-bound cryptoproteins in plasmas from 30 newly diagnosed NSCLC cases (*n* = 3 patients per pool; 10 pools in total) and 102 controls (8–30 individuals per pool; six pools in total) (Supplemental Table S2) [17]. Our analysis yielded 162 Ig-bound cryptoproteins, of which 104 were quantified exclusively in NSCLC case samples (*n* = 90) or had fold-change >2 (*n* = 14) compared to controls (Supplemental Table S4, Figure 2A). Of the 104 cancer-associated, Ig-bound cryptoproteins, 46 (44.2%) were derived from protein-coding genes, 17 (16.3%) from retained introns, 13 (12.5%) from processed transcripts, 7 (6.7%) from antisense RNAs, 10 (9.6%) from lincRNA, 4 (3.8%) from processed pseudogenes, 3 (2.9%) from nonsense mediated decay, two (1.9%) from non-experimentally confirmed regions, and two (1.9%) from sense overlapping transcripts (Figure 2B). Limited overlap was found between Ig-bound, non-canonical cryptoproteins and respective Ig-bound canonical proteins in newly diagnosed cases, suggesting preferential immunogenicity directed against the non-canonical cyptoprotein (Figure 2C). Moreover, 10 out of 104 elevated cryptoproteins were also identified in adenocarcinoma cell lines (Figure 2D).

**Figure 3:**
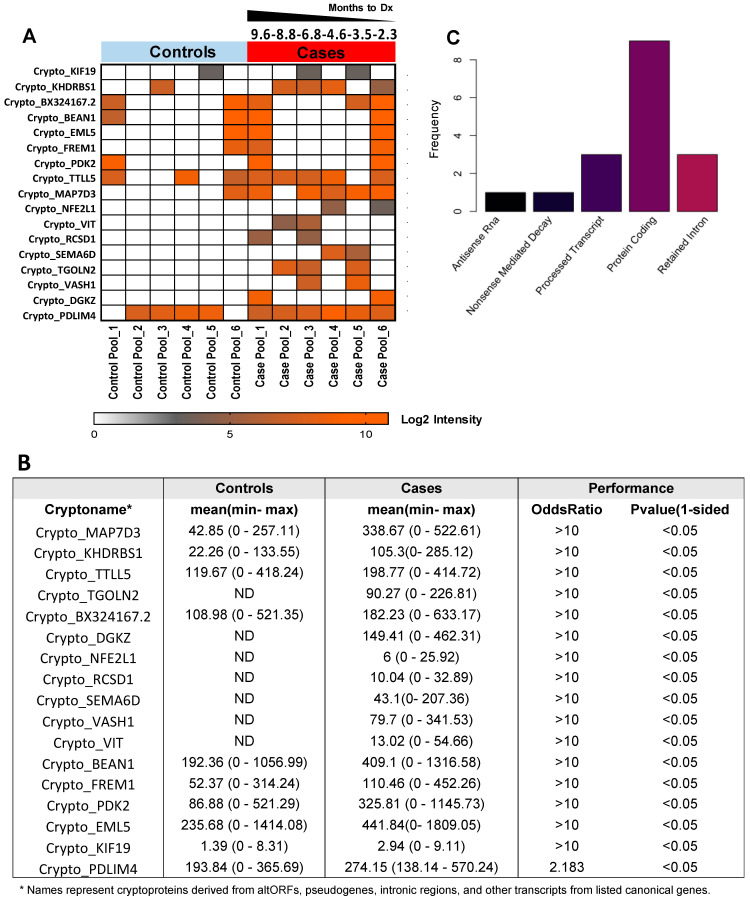
Ig-bound cryptoproteins are detectable in NSCLC pre-diagnostic plasma: (**A**) Heatmap showing 17 Ig-bound cryptoproteins that were elevated (OR > 2; 1-sided *p* value < 0.05) in plasmas collected within 1 year of a lung cancer diagnosis compared to controls. Abbreviations: Sq, Squamous Cell Carcinoma; Adc, Adenocarcinoma. (**B**) Tabular representation of the 17 Ig-bound cryptoproteins. ND = Not Detected. (**C**) Histogram detailing the cryptoprotein type for the 17 Ig-bound cryptoproteins that were elevated in cases diagnosed within 1 year of blood draw compared to controls.

We further performed mass spectrometry-based, Ig-bound cryptoprotein analyses using 6 pools of pre-diagnostic samples consisting of 51 NSCLC subjects diagnosed within an average of six months following blood draw and compared findings with that of the 102 controls (Supplemental Table S3). A total of 101 Ig-bound cryptoproteins were quantified, 17 of which yielded statistically significant (1-sided *p* < 0.05) odds ratio ≥ 2 (Figure 3A,B, Supplemental Table S5). Of the 17 cancer-associated, Ig-bound cryptoproteins, 9 (52.9%) were derived from protein-coding genes, 3 (17.6%) from retained introns, 3 (17.6%) from processed transcripts, 1 (6.0%) from antisense RNAs, and 1 (6.0%) nonsense mediated decay (Figure 3C). Eight Ig-bound cyptoproteins consistently elevated the pre-diagnostic and newly diagnosed cases compared to controls (Supplemental Table S6).

## 3. Discussion

We constructed a cryptoprotein database of theoretical non-canonical proteins that we then applied to search untargeted proteomic datasets of proteomic profiling of lung adenocarcinoma cell lines and patient plasmas. This enabled the discovery of a previously unreported ‘cryptoproteome’ associated with NSCLC and provided evidence of a corresponding humoral response in the form of autoantibodies directed against cancer-associated cryptoproteins. These findings indicate translational potential in the form of candidate markers for early detection or immunotherapy targets.

In contrast to previous studies, which focused on aberrant peptides resulting from genomic alteration [14,15,16], we pursued an untargeted approach for broad non-canonical protein identification unrestricted by mutational status. The cryptoDB provides a resource of possible protein sequences non-homologous to previously described human proteins that can be detected in multiple samples using standard proteomic techniques. This suggests that increased attention may be paid to the protein-encoding potential of RNAs previously thought to be noncoding, and provides potential insights into where these RNAs may alter cellular physiology [25]. We demonstrate application of the database with statistically significant concordant findings across multiple samples, thereby demonstrating the validity of the approach.

Analysis of quantifiable cryptoproteins in newly diagnosed Ig-bound samples, pre-diagnosed Ig-bound samples, and cell lines revealed that quantified cryptoproteins were predominately derived from protein-coding transcripts (>40%), suggesting that the generation of these cryptoproteins is at the translation level. While much emphasis has been placed on proteins resulting from genetic aberrations in cancer, this finding provides compelling evidence of additional routes to the genesis of detectable neoantigens. For instance, this may partially explain the somewhat limited value of tumor mutational burden in predicting response to cancer immunotherapy [26]. An analysis of cell lines provides evidence for this association with translation errors. Several plausible mechanisms may be posited to account for the generation of a specific cryptoprotein, including a “slippery ribosome,” altered nonsense mediated decay, relaxed translational fidelity, or aberrant transcription of ORF-containing pseudogenes [27,28,29,30]. An exploration of potential underlying mechanisms related to the generation of our identified cryptoproteins is warranted; however, such investigations are beyond the scope of the current study.

Interestingly, more than half (56%) of the Ig-bound cryptoproteins were exclusively quantified in NSCLC plasmas compared to controls. Moreover, the ability to detect Ig-bound cryptoproteins in pre-diagnostic plasmas provides the potential that these may be sensitive and specific markers of lung cancer risk and presence of disease. These could either serve to complement to existing markers [31,32,33] or may offer sufficient performance to stand alone as a new source of biomarkers for lung cancer early detection or risk assessment. Future studies, exploring the utility of autoantibodies directed against cancer-associated cryptoproteins alone or in combination with other biomarker types for risk prediction of lung cancer, are warranted.

On the other hand, there are some limitations to our studies. Detailed information regarding full smoking history including smoking duration were not available, thus limiting correlative analyses with smoking exposure. Similarly, the occurrence of cryptoproteins in cancer-associated exosomes or circulating tumor cells was not evaluated [34]. Our study focused on the occurrence of Ig-bound cryptoproteins in plasmas of adenocarcinoma and squamous cell carcinoma lung cancer cases. Whether there is a similar occurrence to be found in small cell lung cancer cases, as well as other NSCLC subtypes, such as large cell carcinoma, remains to be determined.

In conclusion, we establish cryptoproteins as a potential source of neoantigens in NSCLC. Autoantibodies against cancer-associated cryptoproteins are a promising source of biomarkers that may identify individuals at high risk of developing or harboring lung cancer. Future work will include validation in independent datasets, the collection of additional samples, and the biological confirmation of autoantibody reactivity in plasmas of lung cancer patients.

## 4. Materials and Methods

**Construction of a cryptoprotein proteomics pipeline and database**

**Figure 4:**
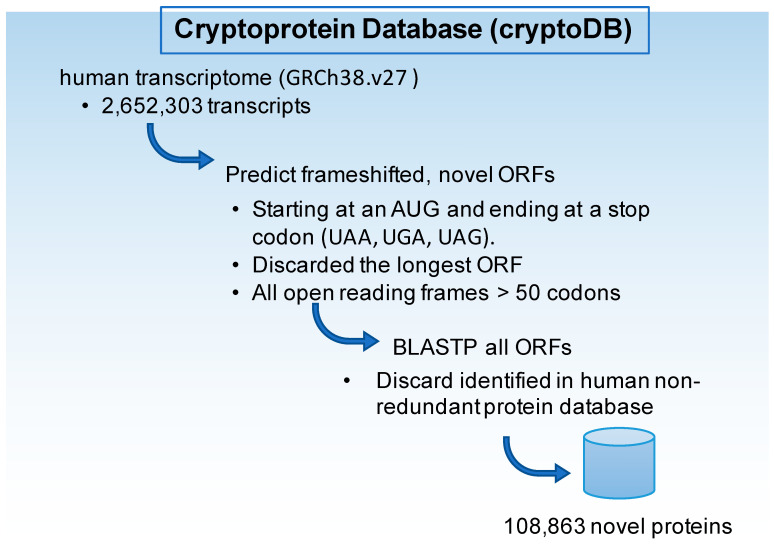
Construction of a cryptoprotein database (cryptoDB).

The fasta format transcript files from Genome Reference Consortium Human Build 38, release 27 (GRCh38.v27), were downloaded from GENCODE. In silico translation was completed for all transcripts including protein coding genes, pseudogenes, noncoding RNAs, including microRNAs (miRNAs) and long noncoding RNAs (lncRNAs), as well as variants including transcripts with retained introns. We selected all open reading frames (ORFs) greater than 50 codons, beginning with an AUG and ending with a canonical stop-codon (UAA, UGA, UAG), and eliminated the largest open reading frame from transcripts annotated as protein coding, which resulted in 1.1 million ORFs. These were then aligned against the human non-redundant (nr) protein database using the BLASTP algorithm. ORFs aligning with an E-score greater than 0.01, indicating a likely successful alignment, were discarded. This yielded 108,863 ORFs with no known homology to human proteins, which we termed the Cryptoprotein Database (cryptoDB, Figure 4). The fasta file of the cryptoDB is available on github (https://github.com/EhsanIrajizad/Cryptoprotein, uploaded April 2021, accessed 10 August 2022).

**Cell Lines**

Seventeen lung adenocarcinoma cell lines (H2228, H1395, H2405, H522, H969, H1703, H1650, HCC827, HCC4006, H820, HCC2935, H2009, H650, H1795, H2122, H647, HCC4017, Supplemental Table S1) were acquired from American Type Culture Collection. Cell type was confirmed by short tandem repeat analysis. The cell lines were representative of different mutational backgrounds and were analyzed independently. Detailed information regarding mass spectrometry-based analysis of whole cell lysates is described elsewhere [35,36,37,38,39].

**Lung cancer plasma collection**

Blood samples were collected from two independent cohorts following Institutional Review Board approval and informed consent. One cohort consisted of plasma collected from individuals with newly diagnosed NSCLC at the University of Texas MD Anderson Cancer Center (MDA) (Supplemental Table S2). Another cohort consisted of plasma samples from individuals collected for the Beta-carotene and Retinol Efficacy Trial (CARET) cohort. CARET was a randomized, double-blind, placebo-controlled trial evaluating the cancer prevention efficacy and the safety of daily supplementation with beta-carotene and retinol palmitate in 18,314 individuals at high risk for lung cancer. Participants were enrolled at six US centers and were followed for cancer and mortality outcomes [40]. Six pools of healthy controls (*n* = 8–28 individuals per pool) were matched to six pools of pre-diagnostic NSCLC cases (*n* = 4–14 patients per pool) based on age, sex, and smoking history. All controls were followed-up for a minimum of four years to ensure that they were cancer-free (Supplemental Table S3). For the MDA cohort, controls from the CARET cohort were used to compare the distribution of Ig-bound cryptoproteins between cases and controls.

**Mass spectrometry analysis of Ig-bound protein complexes**

Mass spectrometry-based (MS) analysis of circulating Ig-bound protein complexes was performed as previously described [36,41]. Detailed has been presented in Supplementary Information.

**Data Processing of Mass Spectrometry Data**

Spectra from proteomic analyses of human lung cancer cell lines and immunoglobulin(Ig)-bound plasma proteins were reprocessed through a customized pipeline based on PeptideShaker [42]. To process only those spectra that did not align to a known UniProt sequence, spectra were first searched against the UniProt Database and spectra identified as UniProt peptides filtered out. Unaligned spectra were subsequently searched against the novel cryptoDB using PeptideShaker [42,43,44,45,46]. We compared the peptide-spectrum match score (PSM) for canonical peptides that matched to the UniProt database to the PSM for cryptopeptides. Peptides were considered a match if they had a false discovery rate (FDR) < 10%, consistent with prior approaches [14]. Furthermore, to reduce false positives, a selection of features in each experiment (cohort) was based on the identified cryptoprotein having a peptide-spectrum match (PSM) ≥ 5 and detection in 2 or more samples in each cohort. Cryptoprotein abundance was implied through summation of all aligned spectra.

**Ingenuity Pathway Analyses**

To identify potential pathway networks associated with cryptoproteins identified in lung adenocarcinoma cell lines, we used host, canonical gene names corresponding to each cryptoprotein, and performed Ingenuity Pathway Enrichment Analysis (IPA). Statistical significance of enriched pathways was determined by two-sided Fisher’s Exact Test.

**Statistical Analysis**

Predictive performance of Ig-bound cryptoprotein complexes was assessed by odds ratios (ORs) using logistic regression for the newly diagnosed cohort and conditional logistic regression for the pre-diagnostic cohort. Analyses were carried out using the R software environment (version 3.6.1, The R Foundation, https://www.r-project.org, accessed 10 August 2022). *p* values are reported based on two-sided Wilcoxon rank sum test unless otherwise specified.

## Data Availability

Other relevant data supporting the findings of this study are available within the Article or are available from the authors upon reasonable request.

## Supplementary Materials

The following supporting information can be downloaded at: https://www.mdpi.com/article/10.3390/ijms23168933/s1.
