# Supplementary material for: A Comprehensive Search of Non-Canonical Proteins in Non-Small Cell Lung Cancer and Their Impact on the Immune Response
Source: Int J Mol Sci. 2022 Aug 11;23(16):8933. doi: 10.3390/ijms23168933 (PMC9409146; doi:10.3390/ijms23168933)
Supplement: Supplementary file 1 [file ijms-23-08933-s001.zip › ijms-1841774-supplementary/Supplementary Information_final.pdf]

Mass spectrometry analysis of Ig-bound protein complexes

Mass spectrometry-based (MS) analysis of circulating Ig-bound protein complexes was performed as previously described.[23,25] Briefly, Ig-bound proteins from a total of 100 μL of plasma were extracted using NAb protein A/G spin columns (Thermo Fisher Scientific) according to the manufacturer's instructions. Columns were equilibrated twice with 400 μL binding buffer (phosphate buffered saline; PBS, pH 7.2) and then incubated for 10 min at room temperature (RT) with plasma samples diluted 1:2 in PBS, pH 7.2. Columns were washed three times with 400 μL of PBS, pH 7.2. Ig-bound proteins were eluted twice with 400 μL of 0.1 M glycine, pH 3. The flow-through was collected and then neutralized with 40 μL of PBS, pH 9. After each step, columns were centrifuged for 1 min at 5000 x g. To reduce non-specific binding to the protein A/G spin columns, an additional low pH wash with 400 μL of PBS, pH 5, was performed before Ig-bound protein elution. For MS analysis, the collected proteins were treated with 25 mM tris(2-carboxyethyl)phosphine for Cys reduction and subsequently alkylated with acrylamide. The samples were next fractionated at the protein level by reverse-phase chromatography followed by desalting for 5 min with 95% mobile phase A (0.1% trifluoroacetic acid (TFA) in 95% water). Proteins were eluted from the column and collected into 12 fractions, with a gradient elution that included an increase from 5% to 70% mobile phase B (0.1% TFA in 95% acetonitrile) over 25 min, 70% to 95% mobile phase B for 3 min, a wash step to hold at 95% mobile phase B for 2 min, followed by a re-equilibration step at 95% mobile phase A for 5 min. For Ig-bound protein analysis, protein digestion and identification by liquid chromatography coupled tandem mass spectrometry (LC-MS/MS), a nanoAcquity ultra performance LC system coupled in-line

with WATERS SYNAPT G2-Si mass spectrometer was used for the separation of pooled digested protein fractions. The system was equipped with a Waters Symmetry C18 nanoAcquity trap-column (180 mm 20 mm, 5 mm) and a Waters HSS-T3 C18 nanoAcquity analytical column (75 mm, 150 mm, 1.8 mm). Data were acquired in resolution mode with SYNAPT G2-Si using Waters Masslynx (version 4.1, SCN 851). The mass spectrometer was operated in V-mode with a typical resolving power of at least 20,000. All analyses were performed using positive mode electrospray ionization using a NanoLockSpray source. The lock mass channel was sampled every minute. Accurate mass LC-HDMSE data were collected in an alternating, low energy (MS) and high energy (MSE) mode of acquisition with mass scan range from m/z 50 to 1800. The spectral acquisition time in each mode was 1.0 s with a 0.1 s inter-scan delay.

Supplemental Table S1. Cell Lines evaluated and number of identified cryptoproteins.

Cell Line	# of cryptoproteins quantified	Known driver mutation
H2228	130	*EML4ALK*
H1395	143	*LKB1*
H2405	126	*TP53*
H522	137	*TP53*
H969	106	*WT*
H1703	123	*TP53*
H1650	122	*WT*
HCC827	147	*TP53, EGFR*
HCC4006	104	*TP53, EGFR*
H820	122	*TP53, EGFR*
HCC2935	127	*TP53, EGFR*
H2009	48	*TP53, KRAS*
H650	27	*TP53, EGFR*
H1795	131	*TP53, KRAS,KEAP1*
H2122	143	*TP53, KRAS,KEAP1,LKB1*
H647	95	*TP53, KRAS,KEAP1,LKB1*
HCC4017	99	*TP53, KRAS*

Supplemental Table S2. Patient and tumor characteristics for newly-diagnosed NSCLC cohort.

Sampling	Subtype	Smoking Status	Smoking pack years (PYs)	Sex	Age	Stage
Pool #1	Adenocarcinoma	Current	75	Female	65	IA
	Adenocarcinoma	Former	40	Female	56	IA
	Adenocarcinoma	Current	6	Female	48	IA
Pool #2	Adenocarcinoma	Current	116	Female	75	IA
	Adenocarcinoma	Current	30	Female	48	IA
	Adenocarcinoma	Former	20	Female	68	IA
Pool #3	Squamous	Former	38	Male	62	IA
	Squamous	Former	75	Female	70	IB
	Squamous	Former	20	Female	76	IB
Pool #4	Squamous	Former	40	Male	73	IA
	Squamous	Former	60	Female	73	IB
	Squamous	Former	40	Female	64	IB
Pool #5	Adenocarcinoma	Former	120	Male	79	IA
	Adenocarcinoma	Former	>100	Female	72	IA
	Adenocarcinoma	Current	40	Male	56	IA
Pool #6	Adenocarcinoma	Former	30	Male	59	IIA
	Adenocarcinoma	Former	30	Female	64	IIA
	Adenocarcinoma	Current	-	Male	59	IIB
Pool #7	Adenocarcinoma	Former	88	Male	61	IIB
	Adenocarcinoma	Current	46	Female	62	IIA
	Adenocarcinoma	Current	>100	Male	71	IIA
Pool #8	Squamous	Current	30	Female	68	IIA
	Squamous	Former	40	Male	68	IIB
	Squamous	Former	75	Male	79	IIB
Pool #9	Squamous	Current	100	Male	60	IIA
	Squamous	Former	-	Male	74	IIB
	Squamous	Current	80	Male	63	IIA
Pool #10	Adenocarcinoma	Former	120	Male	79	IA
	Adenocarcinoma	Current	40	Male	56	IA
	Adenocarcinoma	Former	>100	Female	72	IA

Supplemental Table S3. Patient and tumor characteristics for pre-diagnostic NSCLC cohort.

Sampling	Type	# of individuals	Male/Female	Age (Min-Max)	Current/Former	histology*	stage	Time to diagnosis (months) (average (Min-Max)
**Pool #1**	Case	7	4/3	62 (55-71)	6/1	ADC	I+II	6.8 (1.8-12.4)
**Pool #2**	Case	13	8/7	65 (56-75)	8/5	ADC	III+IV	8.8 (6.1-11.4)
**Pool #3**	Case	12	10/2	64 (54-73)	8/4	ADC	III+IV	3.5 (1.2-5.8)
**Pool #4**	Case	4	2/2	65 (59-70)	3/1	SCC	I+II	4.6 (1.3-9.5)
**Pool #5**	Case	10	9/1	69 (59-77)	5/5	SCC	III+IV	9.6 (7.2-11.3)
**Pool #6**	Case	5	5/0	70 (60-80)	3/2	SCC	III+IV	2.3 (1.8-2.6)
**Pool #1**	Healthy controls	14	8/6	62 (55-71)	12/2	-	-	-
**Pool #2**	Healthy controls	26	16/14	65 (56-75)	16/10	-	-	-
**Pool #3**	Healthy controls	24	20/4	64 (54-73)	16/8	-	-	-
**Pool #4**	Healthy controls	8	4/4	65 (59-70)	6/2	-	-	-
**Pool #5**	Healthy controls	20	18/2	69 (59-77)	10/10	-	-	-
**Pool #6**	Healthy controls	10	10/0	70 (60-80)	6/4	-	-	-

***ADC: Adenocarcinoma; SCC: Squamous cell carcinoma**

Supplemental Table S4: Performance of Ig-bound cryptoproteins in newly diagnosed samples.

See excel file.

Supplemental Table S5: Performance of Ig-bound cryptoproteins in pre-diagnostic samples.

See excel file.

Supplemental Table S6: Overlapping Ig-bound cryptoproteins collected at the time of diagnosis or preceding diagnosis of lung cancer. Eight Ig-bound cryptoproteins consistently elevated in both pre-diagnostic and newly-diagnosed cases compared to controls highlighted in red.

	Newly diagnosed			Pre-diagnostic		
	All cases	Adenocarcinoma	Squamous cell carcinoma cases	All cases	Adenocarcinoma	Squamous cell carcinoma cases
**Cryptoname***	**Fold Change**	**Fold Change**	**Fold Change**	**Odds Ratio**	**Odds Ratio**	**Odds Ratio**
Crypto_KHSRP	7.58	9.42	4.82	1.22	>10	1.11
Crypto_KIF19	5.51	7.41	2.67	>10	>10	-
Crypto_TAF15	3.42	3.09	3.92	0.95	>10	0.05
Crypto_ZFHX3	7.47	-	7.47	>10	>10	-
Crypto_NEU4	1.85	2.06	1.53	0.94	-	0.94
Crypto_PCYT2	1.43	1.87	0.77	0.94	>10	0.91
Crypto_C9orf148	1.19	1.06	1.37	0.80	>10	-
Crypto_TTLL5	1.82	-	1.82	>10	>10	>10
Crypto_OR52U1P	1.13	1.13	-	0.00	0.00	-
Crypto_LINC01358	1.08	1.08	-	1.64	1.40	>10
Crypto_PLVAP	1.52	-	1.52	0.99	0.99	-
Crypto_KHDRBS1	1.21	-	1.21	>10	>10	>10
Crypto_SLC25A4	0.01	0.02	-	1.35	-	1.49

*Names represent cryptoproteins derived from altORFs, pseudogenes, intronic regions, and other transcripts from listed canonical genes.
